# Spiritual Assessment in a Patient With Lung Cancer

**Published:** 2014-11-01

**Authors:** Tami Borneman

**Affiliations:** City of Hope, Duarte, California

## Abstract

**CASE STUDY**

Mr. G., an 82-year-old retired European man, was diagnosed with stage 4 non–small cell lung cancer (NSCLC) and recently enrolled on a phase II clinical trial. He is married and has two adult children, who are very supportive. He and his wife described themselves as nonpracticing Catholics. He had never smoked, and there was no personal or family history of cancer.

Fatigue was the main side effect from the clinical trial drugs, necessitating frequent periods of rest throughout the day and ultimately requiring dose reduction. His left leg was edematous and painful, and he was diagnosed with and treated for deep-vein thrombosis. Over time, these symptoms resolved, and Mr. G. enjoyed a fairly normal quality of life (QOL). He continued to do well for almost a year, but then his cancer progressed and his performance status began to decline. When offered treatment options, he elected to discontinue the clinical trial, take a break, and then initiate single-agent chemotherapy.

Mr. G. was enrolled in a palliative care research study that provided patient-tailored education by an advanced practitioner (AP). The education addressed each QOL domain: physical, psychological, social, and spiritual. When the AP connected with Mr. G. during one of his clinic appointments, he appeared very concerned. He shared that he previously had lived in a communist country and now that he was in the United States, he was afraid of losing his insurance and having to stop treatment. The conversation was interrupted as he was called in for his appointment, yet he consented to talk about the matter further by telephone.

The AP contacted Mr. G. the next day. He shared a glimpse of his childhood and experience in his homeland to try to explain his current fears. After reassuring him that his insurance would not be withdrawn, the AP asked whether he would be willing to talk about his life before coming to the United States more than 50 years ago. She wanted to assess where he was spiritually as a self-described nonpracticing Catholic.

Mr. G. began by stating that he knew he was going to die of his lung cancer. He added that he did not know whether he was afraid of dying or believed in an afterlife, as he felt ambivalent about faith and religion. The AP learned that what gave his life meaning was his family. His "boys" were everything to him, and he did not want to be a burden to them or his wife.

The AP listened and then encouraged Mr. G. to tell his whole story. As a child, he had lived in an occupied country in Eastern Europe during World War II. Mr. G. and his family spent over a year in a concentration camp. They slept on straw, their heads were shaved, and they all had lice. Men aged 18 to 40 were shipped to Russia to work in the copper mines, where many died of exhaustion. Most older men were killed, and he watched his grandfather die beside him. Horse-drawn buggies took dead bodies to mass graves, where lime was poured over them. Mr. G. had boils over his entire body from lack of nutrition. Though technically Catholic, Mr. G. did not ask God to save him; he had seen too much to believe that God would be involved.

One day, he escaped with two other boys. With the help of a stranger, they crossed at night into Romania. They walked for miles into Hungary, where they found shelter in a convent for several weeks. The Mother Superior collected money so he could take the train to Budapest and arranged for him to stay in a Catholic home. From Budapest, he went to Austria, living in refugee camps until moving into an apartment of his own. Mr. G. attended college in Austria and later moved with his wife to the United States, where they raised two boys and owned a successful business.

Lung cancer is the leading cause of cancer death for both men and women. Those diagnosed with localized lung cancer (~15%) have a 5-year survival rate of 54% (American Cancer Society, 2014). For patients with small cell lung cancer, the 5-year survival rate is 6%, and for those with non–small cell lung cancer (NSCLC), it is 18% (Weiland, 2010). However, by the time most patients are diagnosed, they have advanced disease, with 30% in stage 3 and 40% in stage 4 (Lehto, 2014).

In 2004, the National Consensus Project (NCP) for Quality Palliative Care released the first national palliative care guidelines, which comprised eight domains of care (NCP, 2013; Puchalski et al., 2009). Using the guidelines as a framework, the National Quality Forum (NQF, 2006) released 38 preferred practices to implement the NCP guidelines for palliative care and provide a foundation for measuring outcomes. Both the NCP and the NQF determined spirituality to be an essential element of care, as put forth in the fifth domain: Spiritual, Existential, and Religious Concerns.

In 2009, the same year the guidelines were revised, more than 50 interdisciplinary experts met at a consensus conference aimed to create a working definition of spirituality and to provide recommendations to advance the quality of spiritual care in palliative care. The experts concluded, "Spirituality is the aspect of humanity that refers to the way individuals seek and express meaning and purpose and the way they experience their connectedness to the moment, to self, to others, to nature, and to the significant or sacred" (Puchalski et al., 2009).

## THE ROLE OF APs IN SPIRITUAL ASSESSMENT

Including spirituality as part of the overall care of patients is imperative for oncology advanced practitioners (APs). Beneficence is the central ethical principle for nursing, and APs are well positioned to integrate spiritual care into their work setting (Weiland, 2010).

According to the National Organization of Nurse Practitioner Faculties (NONPF), the 2012 amended Nurse Practitioner Core Competencies require spiritual care to be incorporated into the patient’s health care (NONPF, 2012). Likewise, the 2006–2008 Executive Summary on Clinical Nurse Specialist Core Competencies requires clinical nurse specialists (CNSs) to provide competent care that includes spiritual interventions (National Association of CNS, 2008, 2010). The American Nurses Association (ANA) Code of Ethics states, "The measures nurses take to care for the patient enable the patient to live with as much physical, emotional, social, and spiritual well-being as possible" (ANA, 2001). Furthermore, health-care institutions are required by the Joint Commission International to provide quantitative measures regarding spiritual care and services that accommodate the patient’s end-of-life spiritual needs (Joint Commission International, 2013).

## DEFINING SPIRITUAL SCREENING, SPIRITUAL HISTORY, AND SPIRITUAL ASSESSMENT

Addressing patients’ spiritual needs is an overall goal for APs. According to the 2009 Consensus Conference (Puchalski et al., 2009), there is a delineation among spiritual screening, spiritual history, and spiritual assessment.

Spiritual screening involves a quick check for a spiritual crisis that would result in an immediate referral to a chaplain. An example of a relevant screening question would be "Do you have any spiritual beliefs that you would like us to be aware of?" or "Would you like to see a chaplain?"

Taking a spiritual history uses broader questions to gain a better understanding of the patient’s spiritual needs as well as to identify any spiritual distress. It can provide important information on how a patient’s spiritual history may affect his or her ability to cope with the present illness. Anyone involved and responsible for the patient’s treatment plan may conduct a spiritual history (Puchalski & Ferrell, 2010; Puchalski et al., 2009). Examples of spiritual history questions include "What gives your life meaning?" and "Do you have spiritual beliefs that help you cope with stress?" For more spiritual history questions and tools from the literature, see the Table below.

**Table 1:**
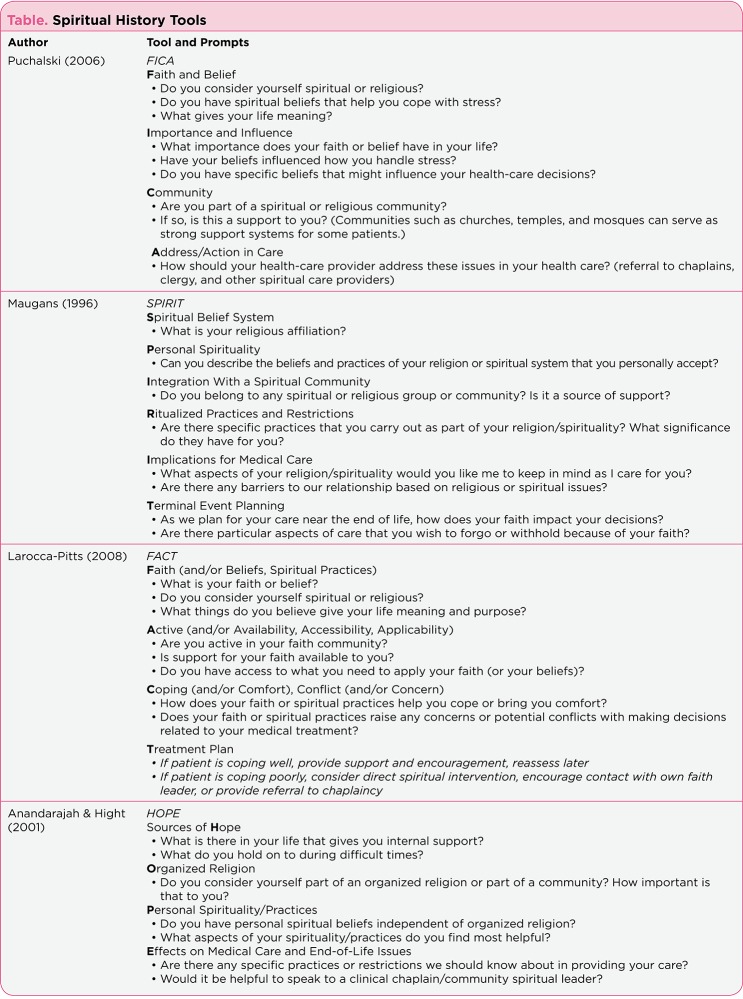
Spiritual History Tools

Ideally, Board-certified chaplains should be the only ones conducting a spiritual assessment, as it is not based on a set of questions. Board-certified chaplains have obtained graduate degrees, are well trained, and are required to complete at least 1,600 hours of clinical pastoral education. Spiritual assessment involves deeper dialog and listening as well as the chaplain’s perception and processing of information containing both objective and interpretive aspects (Fitchett, 2002). However, not all facilities have Board-certified chaplains. In that case, another health-care professional with extensive training and clinical pastoral education may conduct the spiritual assessment.

## CONDUCTING A SPIRITUAL HISTORY: FOCUS ON THE FICA TOOL

The Table provides several tools APs can use to conduct a spiritual history. All of them are short and were created for the busy clinician to provide a good starting point for dialog with patients. The tools can be used with cases of varying complexity yet provide consistency for evaluating patients’ spiritual needs.

In Mr. G.’s case, the FICA tool was utilized. Developed by Puchalski and colleagues in 1996 and later validated in 2010 (Puchalski, 2006; Borneman, Ferrell, & Puchalski, 2010), the FICA tool comprises four domains: **F**aith, **I**mportance of spirituality, **C**ommunity, and **A**ddressing spiritual needs. Advanced practitioners may already know the answers to some of the FICA prompt questions from prior conversations, but going through the tool systematically may reduce the likelihood of missing pertinent information as well as clarifying other information.

**"F": Faith, beliefs, or meaning in life**

*What Is Known*: In the course of Mr. G.’s story, he told the AP that although he is Catholic, he is nonpracticing, so it is more of a cultural belief system than a personal one. He also mentioned that his family members, especially his sons, give his life meaning.

*Possible Prompts*: "How do these relationships help you to cope with your illness?" and "How do your sons in particular give meaning to your life?" The answers to these prompts may provide information that may be helpful with interventions such as leaving a legacy.

*To Think About*: As a young boy, Mr. G.’s life changed in a moment. All that he knew and that had provided security was taken away, and he had watched his grandfather die beside him in a concentration camp. Now he is experiencing stage 4 lung cancer, another traumatic life event. Traumatic life events can destroy a person’s fundamental beliefs about the safety of the world, break close relationships, ruin one’s self-image, weaken one’s belief system, desecrate one’s faith, and propel one into an existential crisis (Herman, 1997).

**"I": The importance of faith or belief in a patient’s life**

*What Is Known*: The AP knows that Mr. G. feels ambivalent about faith and religion and is unsure whether he believes in life after death. Although not much is known about his cultural Catholicism, he stated that in the past, he did not ask God to save him because based on the atrocities he had seen and experienced, God did not seem to be involved.

*Possible Prompts*: "Do your ambivalent feelings toward faith and religion affect how you care for yourself during this illness?"

*To Think About*: In the course of asking prompts and conversing with Mr. G., the AP can look for any signs of anger or resentment toward God.

**"C": Community (religious, spiritual, other)**

*What Is Known*: Mr. G. did not mention friends or a community of support other than his family.

*Possible Prompts*: "In addition to your family, do you feel supported by friends?" "Who would you say makes up your support system?" "Are you supported in any way by those who embrace faith or are engaged in religion?" "Is that of help to you?"

*To Think About*: Mr. G.’s concern over potentially losing his insurance is based on prior experience with socialized medicine. Again, there is the sense of potential loss with the inability to prevent it from happening. A sense of feeling out of control may be an issue for which both a social worker and a chaplain would be beneficial.

**"A": How patients would like health-care professionals to address spiritual needs**

*What Is Known*: Mr. G. did not mention anything about faith or religion beyond his ambivalence to the subject.

*Possible Prompts*: "Mr. G., if you are interested, how would you like us, as your health-care providers, to address your spiritual needs?" "We have chaplains available if you would ever like to talk to one."

*To Think About*: Some patients may welcome a visit by a chaplain, whereas others would prefer to keep their spirituality private. Although Mr. G. was a successful business owner, he learned early in life that he could not rely on others. He had seen too many lives treated as expendable commodities for which there was no protection. As a result, trust may be an issue for him.

## IMPLICATIONS FOR THE AP

Although Mr. G.’s case is neither explicitly spiritual nor common, it demonstrates the complexities of real life and the backgrounds that our patients bring with them. If Mr. G. had simply been asked whether he belonged to a faith community and whether or not he was active in his faith, the AP would not have learned about his time in the concentration camp. It also opened the door for dialog with Mr. G. regarding his spirituality. He ultimately declined a visit from the chaplain, but he was made aware of the available resource.

## SUMMARY

Conducting a spiritual history and providing spiritual care to patients with lung cancer are vital aspects of holistic care. Advanced practitioners should provide care for patients beyond their disease, including addressing aspects of their physical, psychological, social, and spiritual well-being. Ideally, Board-certified chaplains should be the only clinicians conducting a deeper spiritual assessment, but using validated tools such as FICA is a simple way for APs to perform a patient-centered spiritual history to obtain salient information about spiritual concerns, prior spiritual coping methods, and needed spiritual resources.
